# Incubation Period for Neuroinvasive Toscana Virus Infections

**DOI:** 10.3201/eid2712.203172

**Published:** 2021-12

**Authors:** Lison Laroche, Frédéric Jourdain, Nazli Ayhan, Anne-Laure Bañuls, Rémi Charrel, Jorian Prudhomme

**Affiliations:** Université de Montpellier, Institut de Recherche pour le Développement, Centre National de la Recherche Scientifique, Montpellier, France (L. Laroche, F. Jourdain, A.-L. Bañuls, J. Prudhomme);; Santé Publique France, Saint-Maurice, France (F. Jourdain);; Aix-Marseille Université, Institut de Recherche pour le Développement, Institut national de la santé et de la recherche médicale, Marseille, France (N. Ayhan, R. Charrel)

**Keywords:** Toscana virus, sand flies, *Phlebovirus*, imported infectious diseases, arbovirus, meningitis, *Sandfly fever Naples phlebovirus*, *Phenuiviridae*, incubation period, vector-borne infections, Mediterranean

## Abstract

Toscana virus (TOSV) is an emerging pathogen in the Mediterranean area and is neuroinvasive in its most severe form. Basic knowledge on TOSV biology is limited. We conducted a systematic review on travel-related infections to estimate the TOSV incubation period. We estimated the incubation period at 12.1 days.

Toscana virus (TOSV) is an arthropodborne virus transmitted to humans through a bite from an infected sand fly (*1*). An RNA virus, it belongs to the genus *Phlebovirus*, species (*Sandfly fever Naples phlebovirus* family *Phenuiviridae*, order Bunyavirales) (*2*). TOSV infections are endemic to the Mediterranean basin and are considered frequent even though they are neglected (*3*). TOSV can be neuroinvasive and is a major cause of meningitis and encephalitis during summer months in areas to which it is endemic (*4*). However, most infections are asymptomatic or produce mild symptoms (*5*). Thus, TOSV cases are massively underestimated and unreported. Cases are mainly diagnosed by reverse transcription PCR in cerebrospinal fluid, blood, and, rarely, urine or by detecting virus-specific IgM or IgG (*6*). A total of 3 different TOSV lineages (A, B, and C) have been identified, but no clear evidence of a link between clinical manifestation and lineages exists (*7*).

In this study, we considered the incubation period (IP) of an infectious disease as the delay between infection and symptom onset; this definition differs from the latent period, which is defined as the time from infection to infectiousness. For arthropodborne viruses, the infectious bite represents the date of infection (*8*). The potential period of exposure is represented by the length of stay in the country of infection before symptom onset. We therefore focused on imported cases.

Determining the IP is primordial for disease surveillance, outbreak investigation, public health interventions, infectious disease control, and modeling (*9*). However, IP estimates are often unsourced, imprecise, and based on limited evidence, as illustrated by the heterogeneous values proposed (Appendix Table 1). In this context, we conducted a systematic review of symptomatic travel-related neuroinvasive forms of TOSV to provide an evidence-based estimate of the IP.

## The Study

We used PubMed and ISI Web of Knowledge search engines with no restriction on language and the phrase “Toscana AND virus AND (case report OR case-report OR travel* OR import*).” We conducted a systematic search on ProMed and Google Scholar, as well as cross-reference checking. The inclusion criteria were laboratory-documented acute TOSV infection, indication of a travel-related infection in a TOSV-endemic area, and number of days between travel return and symptom onset. Two reviewers screened titles, abstracts, and full-text articles independently.

We extracted clinic and biologic elements from neuroinvasive TOSV case reports. For each patient, data related to the duration of travel and the time of symptom onset, gender, age, country in which case was reported, and country of infection were reported.

To estimate the IP, we used censored time-to-event models (*10*). Interval-censored observations related to travel duration represented the exposure time. Absence of a departure date was treated as left-censored data, whereas onset of illness during the travel period was considered right-censored. We performed data analysis by using R with the icenReg package (R Foundation for Statistical Computing, https://www.r-project.org). We defined the data distribution with 4 parametric models (log-normal, log-logistic, Gamma, and Weibull). To determine the best model for our distribution, we calculated the Akaike information criterion. We used the nonparametric log-rank test from the interval package to assess the effect of age and gender as covariates. To check the result stability, we performed an additional Bayesian approach, fitting with the Weibull distribution.

**Figure 1:**
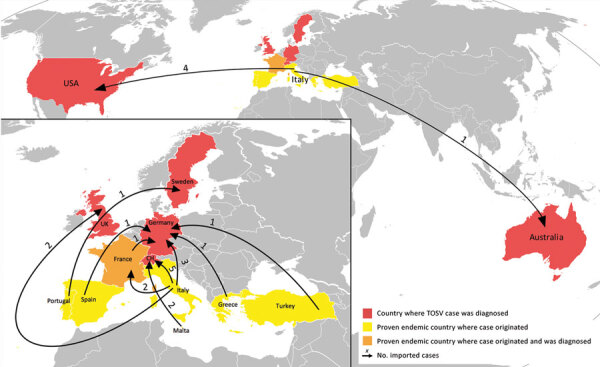
Geographic distribution of imported neuroinvasive cases of TOSV (n = 24) and countries of origin of infection. TOSV, Toscana virus.

Regarding imported case reports, 142 documents were identified on PubMed and 133 on Web of Knowledge. We removed 79 duplicates and excluded 118 records after screening titles and abstracts. A total of 42 articles were eligible after full-text reading. We then selected 22 documents for data extraction. A total of 24 cases were selected (Appendix Table 2, Figure). All travel-associated cases fulfilling the inclusion criteria were neuroinvasive; these cases were diagnosed in a non–TOSV-endemic area after a stay in a proven TOSV-endemic area (Figure 1).

**Figure 2:**
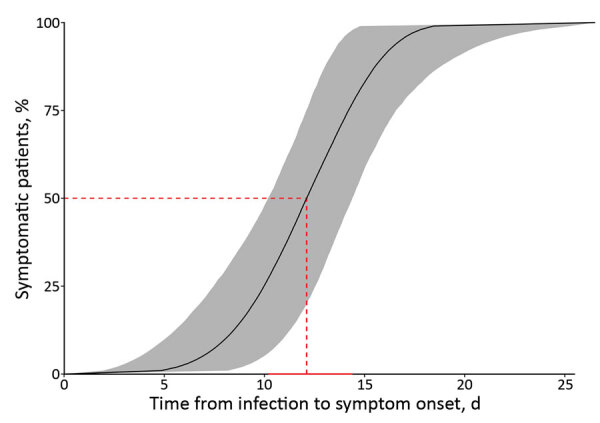
Cumulative percentage of Toscana virus cases manifesting with neurologic symptoms by a given day under the estimates for the Weibull parametric distribution (n = 24). Red dashed line represents the median estimation of the incubation period. Solid red horizontal line represents the 95% CI of the median. Gray shading indicates the 95% CI of the values.

We selected Weibull distribution because it presented the lower Akaike information criterion (Figure 2; Appendix Table 3). The median IP for neuroinvasive forms is estimated to be 12.1 (95% CI 10.2–14.4) days. In 5% of neuroinvasive cases, symptoms will develop by 6.8 (95% CI 3.8–9.9) days after an infectious bite; symptoms will develop in 95% of cases by 16.8 (95% CI 13.9–21.7) days after the infectious bite. We found no evidence of age or gender effect on the length of the IP (p value >0.05 by log-rank test). By using Bayesian analysis, we found an IP of 12.1 (95% CI 9.9–14.4) days (data not shown; results same as Figure 2).

## Conclusions

In the literature, the IP values of TOSV are often heterogeneous, unsourced, or without evidence and therefore do not constitute a valid estimate for clinical or infection control decisions. Our literature review identified 24 neurologic cases of TOSV infection. Some travel durations were reported approximately in case reports and were not included in the analysis. All the data used were based on severe neurologic forms of the disease, which required hospitalization soon after the exposure period.

We estimated the median IP of TOSV at 12 (95% CI 10.2–14.4) days. Considering the delay from infection to symptom onset, this value is greater than that for most other arboviruses (*11*). Our estimate of the IP is evidence-based and relies on data from well-characterized cases. However, cases that cause milder symptoms, as opposed to neuroinvasive forms of the disease, might have a shorter IP (similar to other arboviruses). Other symptoms associated with paucisymptomatic forms of TOSV might not have been described yet and should be further investigated to improve case definition and diagnosis.

We also cannot exclude infections by other sandfly fever Naples phleboviruses because of cross-reaction risk in serologic analyses due to their close genetic relationships (*12*). However, the incidence in the population of other genetically similar phleboviruses is lower than TOSV, and TOSV remains the most common cause of neuroinvasive symptoms (*3*). Knowledge of TOSV genotypes and their aptitude to cause different clinical forms is limited (*12*). Analyzing this hypothesis was not possible because of the limited amount of available data. In addition to the genotype, other parameters may influence the IP, such as viral strain, patient’s immune status, or viremia (*9*). The amount of virus transmitted during bites (viral load) could also influence the IP and should be further investigated.

In addition, all other cases were diagnosed in countries or regions to which TOSV is not endemic (United States, United Kingdom, Sweden, Germany, Switzerland, Australia, and France). These imported cases represent a risk for emergence in these areas when vectors are established (*13*), as has been observed for other vectorborne diseases (*14*). Moreover, sand flies are known to spread in countries or regions to which TOSV is not endemic (*15*).

Currently, information on TOSV infections is lacking (*12*). Precise definitions of the IP should provide more information on the disease epidemiology and on its development in the human host. Moreover, because the IP is a key parameter for disease modeling (*9*), it would improve our understanding of the disease transmission dynamics. More reports of travel-related cases and standardization of data collection with reliable information (e.g., location and duration of the trips and precise dates of symptom onset) are clearly needed. The IP estimation will be improved with addition of new data.

Appendix Additional information about the incubation period for neuroinvasive Toscana virus infections
