# Supplementary material for: Incubation Period for Neuroinvasive Toscana Virus Infections
Source: Emerg Infect Dis. 2021 Dec;27(12):3147–50. doi: 10.3201/eid2712.203172 (PMC8632186; doi:10.3201/eid2712.203172)
Supplement: Appendix — Additional information about the incubation period for neuroinvasive Toscana virus infections [file 20-3172-Techapp-s1.pdf]

Article DOI: https://doi.org/10.3201/eid2712.203172

Incubation Period for Neuroinvasive Toscana Virus Infections

Appendix

Appendix Table 1. Estimation of Toscana virus incubation period described in the published literature

Estimation of incubation period	Year	Reference
≥5 days	1996	(*1*)
From a few days to ≤15 days	1998	(*2*)
From a few days to 2 weeks	2005	(*3*)
3–7 days	2005	(*4*)
3–6 days	2009	(*5*)
A short incubation period	2013	(*6*)
Ranges from 3–6 days to 2 weeks	2011	(*7*)
A short incubation period	2013	(*8*)
3–7 days	2013	(*9*)
A short incubation period	2014	(*10*)
A short incubation period	2014	(*11*)
A short incubation period (2–7 days)	2014	(*12*)
From a few days to 2 weeks	2014	(*13*)
3–7 days	2015	(*14*)
3–7 days	2015	(*15*)
From a few days to 2 weeks	2016	(*16*)
Variable, ranging from a few days to 2–3 weeks	2016	(*17*)
3–6 days	2016	(*18*)
≤2 weeks	2017	(*19*)
3–7 days with a maximum of 2 weeks	2017	(*20*)
From a few days to approximately 2 weeks	2019	(*21*)
Usually 3–7 days	2020	(*22*)

Appendix Table 2. Toscana virus case reports (n = 22) included in the analysis of incubation period estimation*

Reference	Case	Sex	Age, y	Country of infection	Reporting country	Diagnostic methods: Molecular identification	Diagnostic methods: IgM	Diagnostic methods: IgG	Length of stay, d	Time between return and symptom onset, d
(*23*)		M	51	Italy	Australia	+	None	None	17	0
(*24*)		M	66	Italy	United States	–	+	+	16	5
(*25*)		M	61	Italy	Switzerland	+	+	+	21	5
(*26*)†		F	49	Italy	France (Paris)‡	None§	+	+	12	5
(*27*)	1	M	19	France	Germany	None	+	+	14	12
	2	F	73	Greece	Germany	None	+	+	20	12
(*28*)		M	43	Italy	Switzerland	None	+	+	NA	7
(*29*)		M	40	Portugal	Sweden	None	+	+	14	5
(*30*)†		M	68	Italy	France (Paris)‡	None	+	+	11	9
(*31*)		M	17	Italy	Switzerland	+	+	+	21	14
(*32*)		M	82	Italy	United States	None	+	+	14	2
(*33*)		F	69	Italy	Germany	None	+	+	14	−3
(*34*)		M	51	Italy	United States	–	+	+	NA	10
(*35*)		F	18	Italy	United Kingdom	+	–	–	NA	7
(*36*)		M	65	Italy	United States	+	None	None	21	2
(*37*)		F	80	Spain	Germany	–	+	+	NA	4
(*38*)		M	21	Italy	Germany	–	+	+	14	6
(*39*)		M	65	Italy	United Kingdom	+	None	+	4	12
(*40*)		M	34	Turkey	Germany	None	+	+	NA	5
(*41*)	1	M	40	Malta	Switzerland	None	+	+	14	2
	2	F	50	Malta	Switzerland	None	+	+	14	2
(*42*)		M	53	Italy	Germany	None	+	+	10	−4
(*43*)		M	20	Italy	Switzerland	–	+	+	13	1
(*44*)		M	23	Italy	Switzerland	+	+	+	4	10

*NA, no data available; –, negative to Toscana virus; +, positive to Toscana virus.
†Toscana virus case reports diagnosed in Toscana virus–endemic country but in a non-Toscana virus–endemic region.
‡Proven Toscana virus–endemic country but the case was diagnosed in a non-Toscana virus–endemic area of the country.
§Indicates method was not mentioned in the case report.

Appendix Table 3. Incubation estimates, in days, for Toscana virus for 4 competing models (Weibull, Gamma, log-logistic and log-normal) sorted according to the Akaike information criterion*

Model	Median (95% CI)	Log-likelihood	AIC
Weibull	12.1 (10.2–14.4)	−10.16907	24.33814
Gamma	11.5 (9.3–13.9)	−11.26049	26.52098
Log-logistic	11.9 (9.8–14.4)	−11.50064	27.00128
Log-normal	11.3 (9.3–13.9)	−11.78880	27.57760

*AIC, Akaike information criterion.

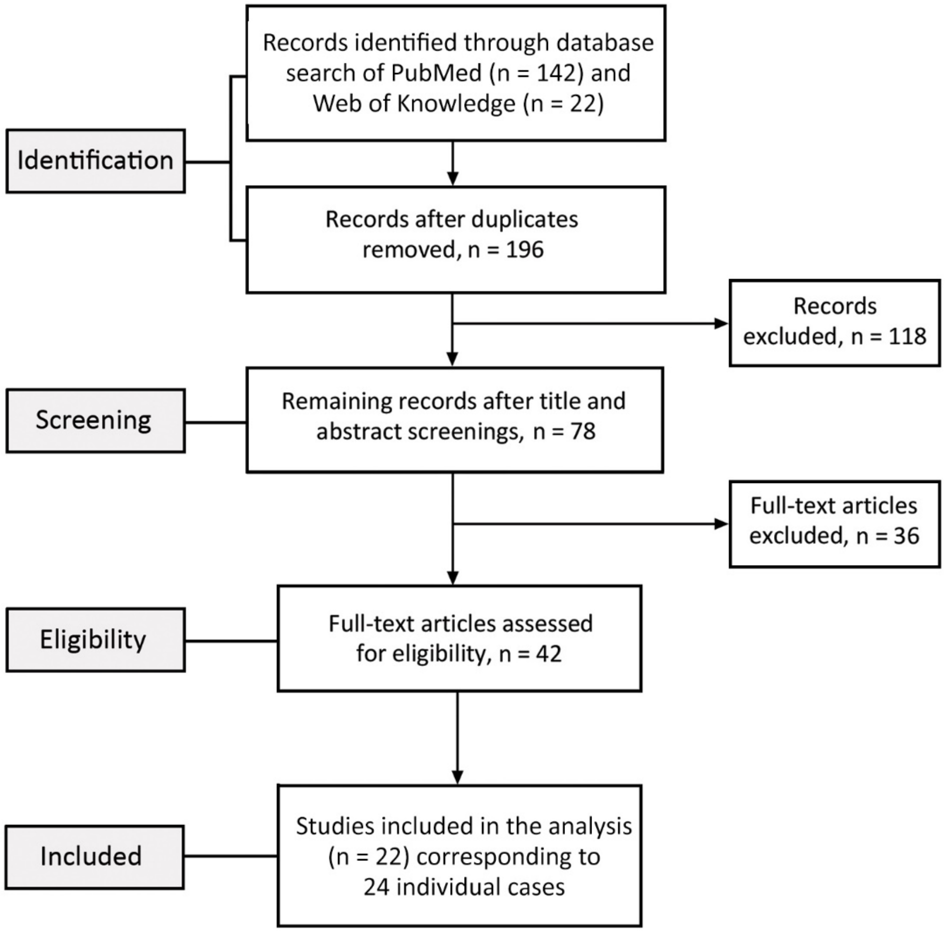

Appendix Figure. PRISMA flow diagram adapted from Moher et al. (*45*) in study of incubation period of Toscana virus

References

1. Nicoletti L, Ciufolini MG, Verani P. Sandfly fever viruses in Italy. In: Schwarz TF, Siegl G, editors. Imported virus infections. Vienna (Austria): Springer; 1996. p. 41–47.

2. Braito A, Corbisiero R, Corradini S, Fiorentini C, Ciufolini MG. Toscana virus infections of the central nervous system in children: a report of 14 cases. J Pediatr. 1998;132:144–8. PubMed https://doi.org/10.1016/S0022-3476(98)70500-1

3. Charrel RN, Gallian P, Navarro-Marí JM, Nicoletti L, Papa A, Sánchez-Seco MP, et al. Emergence of Toscana virus in Europe. Emerg Infect Dis. 2005;11:1657–63. PubMed https://doi.org/10.3201/eid1111.050869

4. Defuentes G, Rapp C, Imbert P, Durand J-P, Debord T. Acute meningitis owing to phlebotomus fever Toscana virus imported to France. J Travel Med. 2005;12:295–6. PubMed https://doi.org/10.2310/7060.2005.12512

5. Sonderegger B, Hächler H, Dobler G, Frei M. Imported aseptic meningitis due to Toscana virus acquired on the island of Elba, Italy, August 2008. Euro Surveill. 2009;14:19079. PubMed https://doi.org/10.2807/ese.14.01.19079-en

6. Nougairede A, Bichaud L, Thiberville SD, Ninove L, Zandotti C, de Lamballerie X, et al. Isolation of Toscana virus from the cerebrospinal fluid of a man with meningitis in Marseille, France, 2010. Vector Borne Zoonotic Dis. 2013;13:685–8. PubMed https://doi.org/10.1089/vbz.2013.1316

7. Serata D, Rapinesi C, Del Casale A, Simonetti A, Mazzarini L, Ambrosi E, et al. Personality changes after Toscana virus (TOSV) encephalitis in a 49-year-old man: A case report. Int J Neurosci. 2011;121:165–9. PubMed https://doi.org/10.3109/00207454.2010.537412

8. Gonen OM, Sacagiu T. Sensory polymyeloradiculopathy associated with Toscana virus infection. J Neurovirol. 2013;19:508–10. PubMed https://doi.org/10.1007/s13365-013-0201-y

9. Anagnostou V, Papa A. Seroprevalence of Toscana virus among residents of Aegean Sea islands, Greece. Travel Med Infect Dis. 2013;11:98–102. PubMed https://doi.org/10.1016/j.tmaid.2012.11.006

10. Dupouey J, Bichaud L, Ninove L, Zandotti C, Thirion-Perrier L, de Lamballerie X, et al. Toscana virus infections: a case series from France. J Infect. 2014;68:290–5. PubMed https://doi.org/10.1016/j.jinf.2013.11.006

11. Magurano F, Baggieri M, Gattuso G, Fortuna C, Remoli ME, Vaccari G, et al. Toscana virus genome stability: data from a meningoencephalitis case in Mantua, Italy. Vector Borne Zoonotic Dis. 2014;14:866–9. PubMed https://doi.org/10.1089/vbz.2014.1668

12. Charrel RN. Toscana virus infection. In: Ergonul O, Can F, Akova F, Madoff L, editors. Emerging infectious diseases. New York: Academic Press; 2014. p. 111–119.

13. Sanchez P, Vázquez González A. Emergence of novel viruses (Toscana, Usutu) in population and climate change. In: Singh SK, editor. Viral infections and global change. New York: John Wiley & Sons; 2014. p. 535–5.

14. Pietrantoni A, Fortuna C, Remoli ME, Ciufolini MG, Superti F. Bovine lactoferrin inhibits Toscana virus infection by binding to heparan sulphate. Viruses. 2015;7:480–95. PubMed https://doi.org/10.3390/v7020480

15. Howell BA, Azar MM, Landry ML, Shaw AC. Toscana virus encephalitis in a traveler returning to the United States. J Clin Microbiol. 2015;53:1445–7. PubMed https://doi.org/10.1128/JCM.03498-14

16. Osborne JC, Khatamzas E, Misbahuddin A, Hart R, Sivaramakrishnan A, Breen DP. Toscana virus encephalitis following a holiday in Sicily. Pract Neurol. 2016;16:139–41. PubMed https://doi.org/10.1136/practneurol-2015-001265

17. Piacenti S, La Ferla L, Capocardo B, Tosto S, Grasso RS, D’Agata V, et al. A case of meningitis caused by Toscana virus. Infect Dis Trop Med. 2016;2:e314.

18. Temocin F, Sari T, Tulek N. Sandfly fever with skin lesions: a case series from Turkey. J Arthropod Borne Dis. 2016;10:608–12. PubMed

19. Veater J, Mehedi F, Cheung CK, Nabarro L, Osborne J, Wong N, et al. Toscana virus meningo-encephalitis: an important differential diagnosis for elderly travellers returning from Mediterranean countries. BMC Geriatr. 2017;17:193. PubMed https://doi.org/10.1186/s12877-017-0593-2

20. Pierro A, Ficarelli S, Ayhan N, Morini S, Raumer L, Bartoletti M, et al. Characterization of antibody response in neuroinvasive infection caused by Toscana virus. Clin Microbiol Infect. 2017;23:868–73. PubMed https://doi.org/10.1016/j.cmi.2017.03.017

21. Tschumi F, Schmutz S, Kufner V, Heider M, Pigny F, Schreiner B, et al. Meningitis and epididymitis caused by Toscana virus infection imported to Switzerland diagnosed by metagenomic sequencing: a case report. BMC Infect Dis. 2019;19:591. PubMed https://doi.org/10.1186/s12879-019-4231-9

22. Ayhan N, Charrel RN. An update on Toscana virus distribution, genetics, medical and diagnostic aspects. Clin Microbiol Infect. 2020;26:1017–23. PubMed https://doi.org/10.1016/j.cmi.2019.12.015

23. Arden KE, Heney C, Shaban B, Nimmo GR, Nissen MD, Sloots TP, et al. Detection of Toscana virus from an adult traveler returning to Australia with encephalitis. J Med Virol. 2017;89:1861–4. PubMed https://doi.org/10.1002/jmv.24839

24. Calisher CH, Weinberg AN, Muth DJ, Lazuick JS. Toscana virus infection in United States citizen returning from Italy. Lancet. 1987;1:165–6. PubMed https://doi.org/10.1016/S0140-6736(87)92005-8

25. Cordey S, Bel M, Petty TJ, Docquier M, Sacco L, Turin L, et al. Toscana virus meningitis case in Switzerland: an example of the ezVIR bioinformatics pipeline utility for the identification of emerging viruses. Clin Microbiol Infect. 2015;21:387.e1–4. PubMed https://doi.org/10.1016/j.cmi.2014.11.010

26. Defuentes G, Rapp C, Imbert P, Durand J-P, Debord T. Acute meningitis owing to *phlebotomus* fever Toscana virus imported to France. J Travel Med. 2005;12:295–6. PubMed https://doi.org/10.2310/7060.2005.12512

27. Dobler G, Treib J, Haass A, Frösner G, Woesner R, Schimrigk K. Toscana virus infection in German travellers returning from the Mediterranean. Infection. 1997;25:325. PubMed https://doi.org/10.1007/BF01720413

28. Dominati A, Sap L, Vora S. [Fever in a returning traveler from Tuscany]. Rev Med Suisse. 2018;14:294–6. PubMed

29. Ehrnst A, Peters CJ, Niklasson B, Svedmyr A, Holmgren B. Neurovirulent Toscana virus (a sandfly fever virus) in Swedish man after visit to Portugal. Lancet. 1985;1:1212–3. PubMed https://doi.org/10.1016/S0140-6736(85)92886-7

30. Epelboin L, Hausfater P, Schuffenecker I, Riou B, Zeller H, Bricaire F, et al. Meningoencephalitis due to Toscana virus in a French traveler returning from central Italy. J Travel Med. 2008;15:361–3. PubMed https://doi.org/10.1111/j.1708-8305.2008.00221.x

31. Gabriel M, Resch C, Günther S, Schmidt-Chanasit J. Toscana virus infection imported from Elba into Switzerland. Emerg Infect Dis. 2010;16:1034–6. PubMed https://doi.org/10.3201/eid1606.091763

32. Howell BA, Azar MM, Landry ML, Shaw AC. Toscana virus encephalitis in a traveler returning to the United States. J Clin Microbiol. 2015;53:1445–7. PubMed https://doi.org/10.1128/JCM.03498-14

33. Imirzalioglu C, Schaller M, Bretzel RG. [Sandfly fever Naples virus (serotype Toscana) infection with meningeal involvement after a vacation in Italy]. Dtsch Med Wochenschr. 2006;131:2838–40. PubMed https://doi.org/10.1055/s-2006-957210

34. Jaijakul S, Arias CA, Hossain M, Arduino RC, Wootton SH, Hasbun R. Toscana meningoencephalitis: a comparison to other viral central nervous system infections. J Clin Virol. 2012;55:204–8. PubMed https://doi.org/10.1016/j.jcv.2012.07.007

35. Karunaratne K, Davies N. Toscana virus meningitis following a holiday in Elba, Italy. Br J Hosp Med (Lond). 2018;79:292. PubMed https://doi.org/10.12968/hmed.2018.79.5.292

36. Kay MK, Gibney KB, Riedo FX, Kosoy OL, Lanciotti RS, Lambert AJ. Toscana virus infection in American traveler returning from Sicily, 2009. Emerg Infect Dis. 2010;16:1498–500. PubMed https://doi.org/10.3201/eid1609.100505

37. Kuhn J, Bewermeyer H, Hartmann-Klosterkoetter U, Emmerich P, Schilling S, Valassina M. Toscana virus causing severe meningoencephalitis in an elderly traveller. J Neurol Neurosurg Psychiatry. 2005;76:1605–6. PubMed https://doi.org/10.1136/jnnp.2004.060863

38. Oechtering J, Petzold GC. Acute hydrocephalus due to impaired CSF resorption in Toscana virus meningoencephalitis. Neurology. 2012;79:829–31. PubMed https://doi.org/10.1212/WNL.0b013e3182661f1a

39. Osborne JC, Khatamzas E, Misbahuddin A, Hart R, Sivaramakrishnan A, Breen DP. Toscana virus encephalitis following a holiday in Sicily. Pract Neurol. 2016;16:139–41. PubMed https://doi.org/10.1136/practneurol-2015-001265

40. Pauli C, Schwarz TF, Meyer CG, Jäger G. [Neurological symptoms after an infection by the sandfly fever virus]. Dtsch Med Wochenschr. 1995;120:1468–72. PubMed https://doi.org/10.1055/s-2008-1055501

41. Schultze D, Korte W, Rafeiner P, Niedrig M. First report of sandfly fever virus infection imported from Malta into Switzerland, October 2011. Euro Surveill. 2012;17:4–6. PubMed https://doi.org/10.2807/ese.17.27.20209-en

42. Schwarz TF, Gilch S, Jäger G. Travel-related Toscana virus infection. Lancet. 1993;342:803–4. PubMed https://doi.org/10.1016/0140-6736(93)91568-7

43. Sonderegger B, Hächler H, Dobler G, Frei M. Imported aseptic meningitis due to Toscana virus acquired on the island of Elba, Italy, August 2008. Euro Surveill. 2009;14:19079. PubMed https://doi.org/10.2807/ese.14.01.19079-en

44. Tschumi F, Schmutz S, Kufner V, Heider M, Pigny F, Schreiner B, et al. Meningitis and epididymitis caused by Toscana virus infection imported to Switzerland diagnosed by metagenomic sequencing: a case report. BMC Infect Dis. 2019;19:591. PubMed https://doi.org/10.1186/s12879-019-4231-9

45. Moher D, Liberati A, Tetzlaff J, Altman DG; PRISMA Group. Preferred reporting items for systematic reviews and meta-analyses: the PRISMA statement. PLoS Med. 2009;6:e1000097. PubMed https://doi.org/10.1371/journal.pmed.1000097
